# The aesthetic values of silence and its impacts on romanticism and contemporary artists

**DOI:** 10.1186/s40064-016-2466-0

**Published:** 2016-06-24

**Authors:** Niloufar Amiri

**Affiliations:** Faculty of Foreign Languages, University of Tehran, Tehran, Iran; US International College Persian School, 871, Alice St., Monterey, CA 93940 USA

**Keywords:** Poetic/living language, Theory of organic unity, Unseen and unheard aspects of language, Metaphoric and ambiguous language, Defamiliarization in language, Modern musical language

## Abstract

In our modern world, where people suffer from self-alienation and are after the meaning of existence in their mechanical and flamboyant outside world, finding a discernible language is very important. People’s dejected minds are the products of miserable modern societies that have changed them into taciturn and uncommunicative creatures in search of meaning. The significance of language, specifically poetic or living language, is undeniable in different eras. Therefore, it would be easier for artists to communicate with people by letting them get the maximum meaning with the least amount of words. This is something that happens in the discourse of modern people. This article shows the aesthetic values of silence and its impacts on romantic and contemporary artists, who for us here will be represented by Samuel Taylor Coleridge as a romantic artist versus Harold Pinter as a contemporary dramatist.

## Background

Language as a means of communication has always been shaped by different circumstances. It is the most powerful instrument of creation enabling us to communicate and connect with the outer world. In different periods, writers, poets, dramatists, and artists in general have always tried to use language in a way that maintains its aesthetic form. For our purposes here, aesthetics means the use of language in accordance with people’s tastes and in line with social and cultural causes. The artistic usage of language has usually glorified its poetic form to give readers pleasure and sometimes to fulfill the artists’ didactic aims. Drama is a genre that allowed, and continues to allow, dramatists to reach both goals.

For instance in romanticism, we witness an elevated language that breaks the boundaries and creates a kind of bliss and ecstasy in readers. The importance of music and its impact on language in this era was so great that poets preferred to choose poetic language—or, better, living language—as the language of drama. The inspiring, sublime, and dynamic language of drama revealed the *unspoken* and *unseen* realities in the society and the world to the audiences of the romantic age.

The radical metamorphosis over time of the poetic language of drama and its modern mutation to nonpoetic language and ultimately silence, in such forms as pauses, have not reduced the aesthetic values of language. That is to say, the techniques used to unveil the disguised beauty of romantic drama still exist in modern drama. This is especially clear in the work of Harold Pinter (1930–2008), the twentieth-century British dramatist known for his so-called comedies of menace, and especially his play *The Dumb Waiter*, which we will examine through the aesthetic theories of the English poet, critic, and philosopher Samuel Taylor Coleridge (1772–1834). A leader of the romantic movement, Coleridge was famous for his theories of language and its importance in drama.

Before considering the aesthetic values of silence, we will first examine some of the conspicuous positions of language in the romantic age and the importance of poetic language both in romanticism, where it was empowered by imagination, and in symbolism. We will review the writings of Andrei Bely and W. B. Yeats on this topic and discuss commonalities shared by romantic and modern artists concerning the significance of language, based on Coleridge’s concept of “organic unity.”

We will attempt to show that although the criteria for the aesthetic in the romantic language of drama may be different from those of modernism, the two share the same goal: to strengthen the image-making power of audiences or readers. It is as if the two are looking at a star from different locations in the world. The locations do not lessen the value and the beauty of that star, no matter how far apart they are.

Language for Pinter, therefore, has the same importance that it does for Coleridge, but his techniques for conveying this meaning to his audiences are different. For his plays, Pinter chooses comedic language, which uses pauses and silences to intensify the humor or deepen the horror of situations. These situations result from the afflictions of men and women in modern society. In fact, when we consider together a number of ideas from Pinter, Coleridge, and other writers like Yeats and Bely, we see that together they amount to the “aesthetic values” of the theater of silence, a theater that despite lacking “elevating language”—as it is traditionally conceived—can be as impressive and effective as romantic drama in its treatment of the dilemmas faced by men and women in the “modern world.” On the stage, silence beautifully turns into the language of modern humanity, conveying different meanings to audiences, as figurative speech did for audiences of the romantic era. Something is “partly missing but it does not let the whole suffer” because the simultaneous presence of language and silence, the two opposites, does not let such a deficiency occur (Coleridge 1983, 30–31). The culture and society of modernity lead its drama to make the great use of pauses and silences. For modern theatergoers, not only is the stage the mirror of society, but the language of the characters should reflect that of ordinary people in their daily lives. The emptiness of language (as the facade of the matter) clearly expresses the emptiness of the characters’ relationships with each other and their boredom in their lives.

My argument views literature primarily as a specialized use of language and proposes a fundamental opposition between the literary use of language in romantic works especially in drama and the ordinary and practical use of language in modern drama. I propose that the central function of ordinary language is to communicate to audiences a message about the world existing outside of language. In contrast, literary language can be seen as self-focused, offering the reader a special mode of experience by drawing attention to its own formal features, such as linguistic signs, which are used in modern drama as a substitute for the rhetorical language of romantic drama. Another aspect of language is the influential formulation of *estrangement* or *defamiliarization* (Abrams 1999, 4), effects achieved by disrupting the modes of ordinary linguistic discourse. Literature makes strange the world of everyday perception and renews the reader’s lost capacity for fresh sensation. Estrangement is effected through techniques deployed by the playwright. For instance, to show the lack of communication from which modern men and women suffer, the playwright fills their attempts at making friendly conversation with repetitions, struggles to find the mot juste, and inarticulation (this last technique used mainly when the character is under great psychological pressure).

We will consider the writings and theories of romantic dramatists on dramatic language and compare the aesthetic values of modern drama to those of traditional drama, particularly as concerns the existence or absence of language.

## Discussion

For both romantic and modern writers, language is our most powerful instrument to communicate and connect with each other and the outer world. For Samuel Taylor Coleridge, language is “the armory of the human mind; and at once contains the trophies of its past, and the weapons of its future concepts” (Coleridge 1983, 2:30–31). It is a dynamic phenomenon, and its dynamism results from the harmony among its particles.

Art for Coleridge is or should be the mediator between man and nature. Poetry is the verbal art capable of establishing such a relation. It has the greatest number of constituent parts (rhythm, meter, musicality, structure, etc.) that bring the highest degree of pleasure by engaging us with a work and enable us to delight in the “whole.” Coleridge believed that every poem or work of art should attain an absolute “organic unity” or “romanticrganicism,” and that all good works of art possess this quality. Through work of the poet, a “synthetic and magical power… reveals itself in the balance or reconciliation of the opposites” (Coleridge 1983, 2:12).

This is what he called “organic unity,” the absolute quality that every poem or work of art should possess. He uses these three elements—(1) the highest degree of pleasure from reading, viewing, or hearing a work of art; (2) becoming engaged in a work of art; and (3) receiving a total delight from the “whole” (Benziger 1951, 24–48)—for the language of drama as well. Coleridge considered the question of the language of verse to be a subset of the larger question of all language. Therefore, he believed that dramatic language should be musical and metaphoric so that it creates what Iranian scholar Bezhad Ghaderi (1384/1996, 82), writing about Coleridge, has called a “tragic dance” in the mind of the reader or audience. The purpose of drama is to produce illusion. Coleridge insists on the active involvement of the spectator or reader in this artistic representation of external nature and human thought and affection (Esterhammer 2006, 144). In this representation we thus have resemblance to the real world but also an element of difference from it. The pleasure we derive from art lies in perceiving “likeness-difference,” identity and contrariety, or what Coleridge terms “the Universal Principle of the Fine Arts” (ibid., 144).

These dual concepts are techniques through which the dramatist creates illusions and pushes the audience to see the “unseen” and hear the “unheard” aspects of language onstage. The Poet or Creator, in ideal perfection, brings the whole soul of humanity into play. He or she diffuses a “spirit of unity” through the power of imagination and balances or reconciles opposite or discordant qualities (ibid., 149).

Such qualities include sameness and difference, generalness and concreteness, familiarity and novelty. Coleridge’s dual and complex thought led him to pursue his aim in an indirect way. He never presented his moral lessons or instructions in a straight line, instead employing techniques that involved the members of the audience, leading them to ponder and ultimately find their mission in their society. He wanted his readers to think, so he wrote in ways designed to encourage and require that effort. For Coleridge, all parts in a work of art, along with the poet and audience (as other particles), should connect with each other so that they achieve harmony and balance. The presence of differences and contrasts notwithstanding, all the particles in a work of art should participate in one unique and harmonized dance.

The modern poets had a more professional approach to language than was then common. There were some influential among modern artists who put the emphasis on the power of language and its symbolic effects like William Butler Yeats (1865–1939) the Irish poet and dramatist, Nobel laureate, and leader of the Irish Renaissance, who still believed in the elevating impact of the poetic language. Here we continue to witness the impact of nineteenth-century poets and writers on some of their twentieth-century counterparts in the powerful influence of language, especially poetic language. Yeats, in an era which life and living came for many to seem meaningless and talking about emotion had lost its real meaning, talks about evoking emotions in a way that Coleridge did before him and in its traditional sense. He finds symbolism as the best remedy for the dying heart of modern man and in the Age of Anxiety. In his article “Symbolism of Poetry” (1937), Yeats writes, “How can the arts overcome the slow dying of men’s hearts that we call the progress of the world, and lay their hands upon men’s heart-strings again, without becoming the garment of religion as in old times?” (Yeats 1980, 28).

In this way, the inactive and gloomy mind of modern man could be activated by the poetic language. Up to this point, we have seen that artists still have the means of “language” in their hands for communication and conveying their messages to the readers. It is true that the language they would use in the twentieth century was different from that of the nineteenth century from different aspects, but it was appropriate for the untidy and cluttered mind of the modern man as well as the chaotic modern world. Stages were the best places to depict the misery, wretchedness and anxiety of man at that time.

Possibly comedy was the best genre to mock the perverseness of modern morality. In this way dramatists would ridicule the groundless standards of modern life as well as convey their messages to stimulate social changes. Some of the artists, like Harold Pinter after Samuel Beckett, started to write laconic, ambiguous symbolic works to show the absurdity of modern man’s life and mind.

Symbolic language still existed in the discourse of modern man but it had lost its rhythm and musicality to arouse reader/audience’s imagination power. The social, economic way of living changed the mind of modern audience and the language of modern men and women were different from that of romanticists. Therefore, we witness the metamorphosis of aesthetics in twentieth-century drama. An ambiguous, fragmentary, and uncertain way of talking was replaced by musicality, rhythm, and a sublime way of speaking, especially in drama.

Applying the above idea to Pinter’s works one could say that he, professionally, by means of the concepts of ambiguity and uncertainty, asks for his audience’s help to find the meaning of his method and the secret of his impact on the stage. He creates this sense of “uncertainty” and “ambiguity” by means of “language” and its absence. Pinter clearly shows that that absent or missing language doesn’t undermine the whole, because the artist, by reconciling the opposites—language and silence—keeps the whole in total balance. This balance enables us to delight not only in language and silence as components but also in the simultaneous presence of these two opposites as a “whole.”

According to Coleridge, artistic representation should create something that resembles the real world but does not copy it (Esterhammer 2006, 144). Efforts to make copies can lead to the end of “imagination” and consequently of “creativity” (Coleridge 1983, 304). Rather than make faithful copies, artists should use ambiguity, or what Coleridge calls “likeness-difference,” to produce “dramatic illusion” (Esterhammer 2006, 145). In this way Coleridge’s artist puts the audience in a state comparable to dreaming but in which the dreamer has more conscious control. Dreams can be reflections of reality, but they are not reality itself. In other words, dramatic illusion is like reality but simultaneously differs from it. Our dreams make us think and interpret them in our own way.

Pinter’s works have the aesthetic quality that all good works of art should possess. His creation can be explained in terms of Coleridge’s theories of language, art, and organic unity. As a dramatist, his use of language creates ambiguity, uncertainty, equivocality, and doubleness. Through these techniques he creates his dramatic illusion and involves his audiences in his works, sending them in search of meaning. In shaping a work of art, Pinter said in a 1962 speech at a drama festival, “a double thing happens. You arrange and you listen, following the clues you leave for yourself, through the characters” (Pinter 1999).

This arrangement, as I quote, lies in careful listening by “tape-recording” the mind, which leads us to shape images and thereby leads us to meaning. Or as Pinter noted elsewhere, “The speech we hear is the indication of that which we don’t hear” (Pinter 1961b, 8–10). Therefore, we need to use our stratagem to discover the core of meaning by discovering or uncovering several layers of words and even silences. As Pinter said in a BBC interview, “Often, below the word spoken, is the thing known and unspoken” (Pinter 1999). Through his techniques Pinter provides us with more questions than answers. He confronts us with afflictions but prescribes no remedy for them.

In pursuing such questions, we could follow romantic writers such as Coleridge and Percy Bysshe Shelley (1792–1822), especially the former’s observation that these answers could be found in the *unheard* and *unseen* (*A Defense of Poetry*, vol. 7, 1965). In Pinter’s case, we are considering specifically the “unspoken” aspect of the dramatic language and also his power of focusing on the “unheard” and “unseen” to his audience by presenting the minimum of language and its juxtaposition to silence in a way that lets them reconcile with each other in a total peace and harmony. The meaning in Pinter’s works lies in unspeakables and even unseens. To apprehend it, we need attentive ears and perceptive minds. Pinter’s works could be conceived of as scrambled puzzles. That is to say, the playwright gives us some pieces but not all of them. The missing pieces are the ones he creates through his techniques of word games, repetition, speechlessness, silences, and pauses. The audience’s active participation is required to find the missing pieces. By “arranging to listen” and by hearing the unheards and seeing the unseens, the audience makes the puzzle complete. By resisting mentioning speakables, Pinter expertly moves us toward unspeakables. As he remarked, “I’m aware sometimes, of an insistence in my mind. Images, characters, insisting upon being written” (quoted in Blanchard 2001).

Here, the constituent parts need to be processed based on organic unity, without which conformity is impossible. All the particles, including the dramatist, his techniques, and the active audience, labor hand in hand to make a harmonized work of art. The process takes place either during the course of a performance or one of reading or after finishing a performance or reading. Let us explore each scenario in greater depth.

In the first, the audience or the reader is supplied with the language and its related techniques. Ambiguity and duality are in fact the essence of life for modern men and women. When confronted with these dualities, readers or audience members are confused at first. A kind of brainstorming occurs in their minds, but when it goes further they adapt themselves by revising their previous thoughts. In this mode of involvement in the creation of meaning, the audience or reader goes half of the way.

What happens in the creation of meaning *after* a performance or reading is not separate from meaning-making *during* it. In fact, the two are interconnected. The perception takes shape thanks to the “tape” that has been recorded during the performance or reading. Imagination gets involved when the audience or reader needs to find the “missing pieces of the puzzle.” This leads them to go the second half of the way in the creation of meaning. Of course, the trend of perception can differ with each reading or performance. The listener or reader does not always have time to go the whole way and reach his or her closure point. Although the fundamental structure of the work of art provided by the artist is the same for all audiences or readers, the ways to make meaning or the mechanisms of perception vary from individual to individual. Lack of attention, passive involvement, or poor listening can cause the missing piece of the puzzle to die and the “whole” to become merely an incomplete and disharmonized artifact.

In both cases, as I quote again, the audience’s image-making power emerges out of a “wrestling in the mind”. With the help of these images, the audience fills in the gaps and puts the missing pieces in the blank areas marked by ambiguous or uncertain silences and pauses.

One of the ways Pinter creates the moment of uncertainty is through the “comedy of menace,” an important technique in fashioning dramatic illusion. Through menace, Pinter reveals unexpected moments of human alienation and isolation. In microcosm (onstage) funny issues become horrifying and disturbing in macrocosm (real life), turning comedy to menace. This alliance—like Pinter’s other techniques, such as silence, dramatic uncertainty, and repetition—terrifies us. We, as audience, attempt to fill in the blanks with our own interpretations, leading us to ponder and confront unknowabilities, since we cannot assume any notions, having only their facades. In this, Pinter, just like a surgeon dissects a corpse before his students without thorough explanation of his performance. It is up to the professor’s audience to diagnose the malignancy in the corpse. The corpse in Pinter’s works could be interpreted as political issues, human frustrations, alliances, and so on.

Pinter’s awareness of the fragility of human relationships and communication, as well as his knowledge of everyday human life, takes form onstage. His artistic representation has portrayed external nature and human thoughts. Although his plays use the language of ordinary people, his language of silences and pauses takes his works beyond the mundane to what Coleridge would call the “particular mode of representation” that proceeds by “imitating not copying” (Coleridge 1983, 154).

Drawing a sharp line between language and silence in Pinter’s works seems impractical because it is through silence that characters communicate about what is “unsaid,” and conversely it is through language that they speak about the “unheards.” In his works, language and silence are not mutually exclusive; each is an intrinsic part of the other. Silence is the language of his characters, and it is up to the audience or reader to decode the meaning of the mute language onstage. Here the audience’s active participation becomes essential. The presence of Pinter’s innovative language next to silence, and the reconciliation of language and silence along with the cooperation of the whole particles, gives us a unified work of art whose mystification by us as actively involve audiences, providing us with a kind of pleasure and ecstasy. Pinter knows how to “imitate” the silence of life through his techniques and characters and their silences and pauses. In this way we have the emergence of Coleridge’s particular mode of representation that is “an imitation and not a copying.” The imitation of real life is conspicuous in Pinter’s drama, *The Dumb Waiter*, where we witness the confrontation of the two worlds of “man and his society.” Although the two seem to be opposites, however, they are reconciled in that one makes the other. This notion runs through both plays. The points where Pinter chooses to show this are usually “far from the madding crowd,” moments when the notion of silence is magnified and the characters can better face the depth of human nature as well as their own frustrations and complexities.

Perhaps we could compare the isolated places of the characters’ microcosm with the very idea of “solitude” in romanticism. The main artistic goal in the two eras (romanticism and modernism/postmodernism) is to enhance contemplation and meditation by readers and audiences by confronting them with silence or with being in solitude. For instance, Gus and Ben, the two characters in *The Dumb Waiter*, are professional assassins working for an organization and getting their orders from the outside world. In fact, they have isolated themselves in the microcosm and their fear of somebody or something in the macrocosm. Many elements in the play create moments of fear, menace, and uncertainty in both the characters and the audience. This anxiety is mainly rooted in the characters’ and the audiences’ ignorance of the unknown. They do not know what is going to happen exactly, or where it will occur. The presence of another door, a paneled opening into the dark, menacing outside world, intensifies the feeling of isolation and menace.

The ambience of isolation and Pinter’s other techniques of deliberate silences and pauses have multifunctional results and can be illuminated by Coleridge’s theory of organic unity. In *The Dumb Waiter*, we have the “presence” of an envelope “without any word.” Here a “double thing” has occurred. This is another clue by which the artist activates his audience members to fill in the gaps with their own pieces of the puzzle that emerge from their imagination. Menace takes place through “unspeakables”: what should a letter without a message convey? Here we have the “presence” of a letter and the “absence” of words, but also the latter’s simultaneous presence as Pinter pushes us toward a feeling of alliance and uncertainty, which he sometimes intensifies with silences or pauses. The envelope scene starts with a “pause” that conveys a threat, a moment of nonverbal tension resulting from the ignorance of knowing nothing. All these techniques jointly create a kind of “brainstorm” in the minds of spectators, in spite of all “dualities” and “ambiguities.” In the following scene from *The Dumb Waiter*, the characters Gus and Ben receive a letter:

*Pause. An envelope slides under the door, right. GUS sees it. He stands, looking at it.*

GUS. BEN.

BEN. Away. They’re all playing away.

GUS. BEN, look here.

BEN. What?

GUS. Look.

*BEN turns his head and sees the envelope. He stands.*

BEN. What’s that?

GUS. I don’t know.

BEN. Where did it come from?

GUS. Under the door.

BEN. Well, what is it?

GUS. I don’t know.

*They stare at it.*

*It continues until they open the envelope.*

*GUS opens it and looks inside*.

What’s in it?

*GUS empties twelve matches into his hand.*

GUS. Matches.

BEN. Matches.

GUS. Yes.

BEN. Show it to me.

*GUS passes the envelope. BEN examines it*.

Nothing on it. Not a word. (Pinter 1961a, 95–96)

In another scene, the presence of a speaking-tube next to the dumb waiter intensifies their desire to communicate with the “unseens.” Metaphorically, we could say that Pinter, by placing a “speaking” tube next to the “dumb” waiter (two opposites), wants to show the human need for communication.

*GUS, turning for a pencil, suddenly discovers the speaking*-*tube, which hangs on the right wall of the hatch facing his bed.*

GUS. What’s this?

BEN. What?

GUS. This.

BEN (examining it). This? It’s a speaking-tube.

GUS. How long has that been there?

BEN. Just the job. We should have used it before, instead of shouting up there.

GUS. Funny I never noticed it before.

BEN. Well, come on.

GUS. What do you do?

BEN. See that? That’s a whistle.

GUS. What? This?

BEN. Yes, take it out. Pull it out.

Gus does so.

That’s it.

GUS. What do we do now?

BEN. Blow into it.

GUS. Blow?

BEN. It whistles up there if you blow. Then they know you want to speak. Blow.

GUS. Blows. Silence.

GUS (tube at mouth). I can’t hear a thing.

BEN. Now you speak! Speak into it!

*GUS looks at BEN, then speaks into the tube.* (Pinter 1961a, 111)

Such scenes give the audience pieces of an incomplete puzzle. The spectators, together with the characters, encounter the “unknown,” which is unseen as well. Pinter thus invites the audience to imagine who or what might be on the other side of the tube, another piece of puzzle. The reconciliation of listening and speaking in the scene helps Gus and the audience face the unknown and overcome their sense of alienation and isolation by striking up communication with the macrocosm. The cooperation of the spectators, through their senses, and the characters, through techniques designed by the playwright, gives a smooth flow to the process of the play. The spectators, like the characters, learn how to remove this dramatic illusion and ready themselves to complete the puzzle.

This is another way of considering the aesthetics in Pinter’s works. All his effort is to make us contemplate; he does this by creating “solitude” for characters and audiences, and by using the language of nature: silence. Again we see here the link between Pinter and Coleridge.

Pinter makes ordinary language and the routine of daily life seem strange. Through the *defamiliarization* of language, he makes ordinary discourse different. Perhaps this defamiliarization is characteristic of the ambiguous and equivocal mind of modern men and women, who require something similar to ourselves: ambiguous language, painting, drama, poetry, and art in general. Our tendency, in spite of ourselves, is to relate and communicate in way that requires a kind of language near to our uncertain way of thinking, living, and speaking. Writers and dramatists of our age should convey “meaning” in a form appropriate to the distorted modern mind and our mechanical society. Artists have found themselves responsible for reviving the dying souls of modern people by uncovering the “unseen” and “unheard” around them and in their societies.

Here will I couch my limbs,

Close by this river, in this silent shade,

As safe and sacred from the step of man

As an invisible world – unheard, unseen

Thomas G. West writes that for Yeats, in his famous essay “The Symbolism of Poetry,” “the only way to overcome the slow dying of men’s heart in the modern society is through the symbolism of poetry and that we needed to return to imagination through the symbolisms [that] existed in poetry in which the laws of art were the hidden laws of the world. If the laws of art could provide us with pleasure, therefore the hidden laws of the world, which was the same as art, could make our life pleasing and as a result meaningful” (, 21). According to Yeats, West writes, “the imagination which could be evoked by *living language* consequently evokes our emotions and this has been something that the modern people have needed to possess” (22).

Years after Yeats’s “Symbolism and Poetry,” Russian symbolist novelist Andrei Bely articulated his idea about living language, which was in line with Yeats’s. In his article “The Magic of Words,” Bely called living language a condition for the existence of mankind itself. He argued that “the purpose of communication is to kindle the signs of communications, i.e. the words with the fire of ever new creative processes. The purpose of living communication is the striving towards the future…. living language is an eternally flowing, creative activity, which raises before us a series of images and myths; our consciousness derives power and confidence from these images” (Bely 1980, 127).

The significance of living language for artists such as Coleridge and later Yeats and Bely results from its image-making power. In our mechanical modern age the existence of creativity, which results from the image-making power of living language, is essential. This is why artists like Harold Pinter, artists aware of the significance of this need, try to include this type of language in their artifacts in a way that is perceivable and near to the everyday language of the people of their time. But perhaps the living language for modern artists like Pinter is not directly poetical. It seems that he applies an indirect poetical language, one that could consist of all the elements of a living language and that has at its core image-making power.

This indirect poetical language could help a modern dramatist like Pinter achieve his goal according to the tastes of the modern people of his time. Poetic plays make metaphors and persuade the audience to play essential dramatic games of presence-absence, likeness-difference, identity and contrariety, and so on. A poetic play does not entail the mere use of poetry in the literary sense; instead, it is a play that achieves metaphorical strength through words, actions, visual images, and musicality, one that bears the same qualities noted by great artists of previous centuries but in a modern way. The words of T. S. Eliot (1888–1996), in his essay “Poetry and Drama,” could be helpful in this regard: “A verse play is not a play done into verse, but a different kind of play…. the poet with ambitions of the theater must discover the laws, both of another kind of verse and of another kind of drama” (Eliot 1994, 145).

The musicality of modern language possesses the characteristics of the music of our age. John Cage, the American experimental composer and leading figure of avant-gardism for more than half a century, predicted the use of noise (both intentional and unintentional) and the sound of everyday objects as well as electronically produced sounds in music. “His work from this period (1900s) is among the first to give noise equal status with musical tone.” He came to believe that music should “imitate nature in her manner of operation.” (Microsoft Encarta Premium 2006).

Since “nature” plays such an important role in our perception of life, artists like Cage find it essential for modern people to focus on (or imitate, as Coleridge would put it) the way nature operates, and Cage tried to do this in his music. Nature operates “in a silent way.” The “silence” of nature is full of “meaning”; instead of being absorbed in our mechanical lives, we have a chance in “silence” to “contemplate” and as a result imitate (not copy) nature’s manner in a powerful creative way, a process that helps us improve our “hearing” as well as “seeing” senses.

The goals of modern art in forms such as drama and poetry is not dissimilar to the aspirations artists of previous eras formulated. Both groups of artists need to communicate with people in their own language, taking into account their psychological, social, and political requirements. Amid the bombardment of our senses by the mechanical, deceitful world, we need interruption zones, noticeable pauses and silences.

Cage used such interruption zones to communicate with people in the language of nature and allow them to concentrate on the “unheard” aspects of happenings, events, and actions around them. The most famous example is his *4′33″* (1952), a silent piece lasting 4 min, 33 s, which elevates incidental, unintended noise in the concert hall to the status of art. Instead of a musical performance, the piece offers “silence,” the language of modern humanity. It is in “silence” that meaning is found, and while we are in silence, we have something to say. As Cage stated in one of his poems, “I have nothing to say, I am saying it, and that is poetry” (Cage 1959, 109).

For Cage, the poetical/musical language, with its economy or absence of words, conveys the most meaning. The “doubleness” in the music of our age exists in our language as well. It seems that terms such as *ambiguity*, *doubleness*, *equivocality*, and *vagueness* are intrinsic to our literature, philosophy, and other kinds of art. Dramatists such as Harold Pinter have been thoroughly aware of the ways modern men and women communicate, and the ways artists should communicate with them. His tactics in establishing communication are so comprehensive that it seems he is echoing all the techniques of previous artists. He knows the unique strength of theater, the imagination encouraged by a live performance. Great plays have always been and will always be poetic plays with “living language.”

## Conclusion

As we have seen, despite the centuries intervening between the careers of Samuel Taylor Coleridge and Harold Pinter, the latter’s plays possess the aesthetic values that the former considered in his theory of “organic unity” in all kinds of works of art universally. In other words, the passage of time and the emergence of new thoughts and philosophies do not necessarily entail rejecting or digressing from what previous artists and aestheticians believed. There is nonetheless a difference, not in the philosophies and theories of art but in the ways artists try to bring to light their artifacts for their audiences or readers. One of their modes for creating a work of art is by making a connection with the people of their time.

People’s ways of thinking differ from era to era and from generation to generation. Our focus here has been on the “psychological differences” that are influenced by social, political, and cultural issues and the major changes we observe in all aspects of life from the nineteenth century on. As René Wellek observed, “The Industrial Revolution, which had begun in England in the eighteenth century, spread over the Continent and transformed living condition radically. The social and economic changes were closely bound up with shifts in the prevailing outlooks and philosophies” (Wellek 2001, 713).

Industrialization, wars, economic change, mechanization, and a focus on the flamboyant outside world have led people far from their inner selves, leaving them to search for their identities and the meaning of their unfamiliar true nature. In this chaotic situation, it is up to artists to depict reality and lead people back to themselves and their societies. Living in this ambiguous and uncertain world and being perceivable by others requires that we have our own language. The role of artists has been to revive the dying and failing souls of modern people by uncovering the “unseen” and “unheard” notions around us.

Yeats’s belief that the artist must “overcome the slow dying of men’s hearts that we call the progress of the world” was not that different from Andrei Bely’s view of “living language.” But what is the role of “living language” in an absurd world? What exists in the “living word” that ordinary language lacks? The answer lies in the image-making power of living or poetic language. As Bely wrote in “The Magic of Words,” “living language is a condition for the existence of mankind itself; this condition is the quintessence of mankind itself; and this is why poetry, knowledge, music and speech were at first a unity; and this is why living language was magic, and why people who spoke such a language were impressed with stamp of communion with deity itself” (Bely 1980, 124).

For Bely, and for most artists, the worth of language lies “the purpose of communication,” which is to kindle the signs of communication, i.e. the words, with the fire of ever new creative processes. The purpose of living communication is the striving towards the future…. living language is an eternally flowing, creative activity, which raises before us a series of images and myths; our consciousness derives power and confidence from these images” (ibid., 126–27).

In our mechanical age, the creativity that results from this image-making power of living language is essential. That is why artists like Harold Pinter include this type of language in their artifacts in a way that is perceivable and near to the everyday language of the people of their time. Wordplay stands in the same place as communication does. As Bely adds, “Language is the very creation of living relationships. If word-play has no purpose, then we adopt a purely aesthetic point of view; but when we realize that aesthetics is only a facet, which refracts the creation of life in its own way, and, in itself, beyond this creation, has no part to play, then the purposeless play with words turns out to be full of meaning: a combination of words, irrespective of their logical meaning, is the means by which man defends himself from the pressure of the unknown” (ibid., 130).

The meaningfulness of wordplay has not faded over the past half century. As we have observed, it was a vital technique in Pinter’s drama. Fear of “unknowns” is one of the motivations that render Pinter’s characters silent and sometimes speechless. This is also why they prefer to resort to wordplays and indirection.

The stage (as microcosm of society and its afflictions) remains the best place to depict people’s hardships and miseries, as well as to make people aware of their situation and what is happening around them.

To the scholar, perhaps “indirect poetical language” could consist of all the elements a “living language” should possess, and the pleasure it provides results from its “image-making” core, which shows the commonalities between artists of the present and the past. The artists (and especially, for our purposes here, the dramatists) of our age need to use both poetic and nonpoetic language to preserve its multifunctional tasks, such as image-making, provoking the emotions, and stimulating active participation by the audience.

We could say that this is the approach followed by most modern dramatists, especially if we believe that only poetic plays make metaphors and persuade the audience to play essential dramatic games of presence-absence, make-believe, likeness-difference, and so on. By a “poetic play,” I do not mean one that merely uses poetry in the literary sense. Rather, I mean one that achieves metaphorical strength through words, actions, visual images, musicality, subtext, and so forth, which have the same qualities cited by prominent artists in previous centuries but convey them in a modern way.

Applying the notions we have discussed here, especially organic theory, to Pinter, we can say that the stage is a metaphor. His form is complex and has been intensely studied. He can be a very lyrical writer in his treatment of memory and at the same time a funny writer when he makes us laugh (bitterly) at our afflictions. But above all, his form allows him to explore the instinctive antagonism that characterizes human relations. We fight each other not with words alone but with “words and silences.” He celebrates the ambiguity and lets the audience wrestle with the contradictions and oppositions he deliberately places in his dramas.

These oppositions keep his work “open,” and this “openness” gives his works a sense of beginning rather than closure. The ambiguous music of modernity, which is based on people’s thoughts and tastes, takes the form of ambiguous “indirect musical language” in his dramas to provoke audiences’ imaginative power. The musicality of his language is found in its combination with “silence.” The peaceful reconciliation of these two opposites makes his works aesthetically perfect. Language finds no meaning in the absence of words, and silence is strengthened by the presence of language.

The dual nature of Pinter’s work, the simultaneous coexistence of opposites within it, has an impact on the audience. The amusement, or let us say the pleasure, of the accuracy of observation, on the one hand, combined at a deeper level with the unease, the mixture of horror and fascination evoked subconsciously, on the other hand, makes this sense of duality complete. We may be amused by the “blind man’s buff” (Pinter 1960) game on the surface, but deep down we sense that this amusement also expresses some contempt and fear.

All uncertainties, ambivalences, and ambiguities of plot and language in Pinter’s plays are expressions of a genuine perplexity about the nature of our experiences of the world. They constitute a carefully recorded creative process, and they will surely endure as considerable artistic achievements. Pinter’s creation of harmonious, congruous works with disharmonious and incongruous artistic elements speaks to audiences in their own language. With the “absence of words” he makes connections with modern people who are totally familiar with this type of short and digressive language. He expertly lets the “opposites” reconcile in his works and keeps the “whole” in complete balance while there is absence of language or presence of silence. This is exactly what Cage does with his music. In this way modern artists and musician such as Pinter and Cage both echo Coleridge’s theory of organic unity by applying the “simultaneous presence of language and silence” all through their works, giving them universal aesthetic values. We could say, then, that they simply reinforce what their artistic predecessors believed, creating and innovating a kind of poetical/living language as the language of their works in a modern way and for the modern people of their time. In other words, we could say that contemporary artists, and especially dramatists, by echoing Coleridge’s theory of “organic unity” and applying the “simultaneous presence of language and silence,” give their works universal aesthetic values. As the Zen koan reminds us, it is the silence between the notes that makes the music.
